# Polishing De Novo Nanopore Assemblies of Bacteria and Eukaryotes With FMLRC2

**DOI:** 10.1093/molbev/msad048

**Published:** 2023-03-03

**Authors:** Q X Charles Mak, Ryan R Wick, James Matthew Holt, Jeremy R Wang

**Affiliations:** Department of Biological Sciences, National University of Singapore, Singapore, Singapore; Centre for Pathogen Genomics, University of Melbourne, Melbourne, Australia; Pacific Biosciences, Inc., Menlo Park, CA, USA; Department of Genetics, University of North Carolina at Chapel Hill, Chapel Hill, NC, USA

**Keywords:** de novo assembly, polishing, nanopore

## Abstract

As the accuracy and throughput of nanopore sequencing improve, it is increasingly common to perform long-read first de novo genome assemblies followed by polishing with accurate short reads. We briefly introduce FMLRC2, the successor to the original FM-index Long Read Corrector (FMLRC), and illustrate its performance as a fast and accurate de novo assembly polisher for both bacterial and eukaryotic genomes.

## Introduction

Long-read, third-generation sequencing technologies including Oxford Nanopore Technologies (ONT) and Pacific Biosciences (PacBio) are increasingly the workhorse and backbone of de novo genome assemblies (Kim et al. 2021; Pollard et al. 2018; Amarasinghe et al. 2020). Median read lengths from 10 to 100 s of kilobases are routinely achieved (Shi et al. 2016; Michael et al. 2018), with which modern assemblers produce much more contiguous and complete de novo assemblies than those from short-read next-generation sequencing (NGS) alone (Pollard et al. 2018; Amarasinghe et al. 2020). Despite continuous improvement in nucleotide-level accuracy of long-read sequencing, residual errors—both single-nucleotide mismatches and short insertions and deletions (indels)—still exceed short-read sequencing-by-synthesis technologies (Pollard et al. 2018; Amarasinghe et al. 2020). Residual consensus errors in long-read assemblies are dominated by indels which hinder gene annotation (Watson and Warr 2019). A “hybrid” assembly approach is commonly taken to maximize assembly accuracy and contiguity by first producing a draft assembly from long-read sequences, followed by polishing with accurate short reads (Jain et al. 2018).

FM-index Long Read Corrector (FMLRC; Wang et al. 2018) is a hybrid error-correction method that employs a Full-text Minute-space (FM) index of a Burrows–Wheeler transform (BWT) built from accurate reads to dynamically reassemble erroneous subregions of error-prone long sequences. FMLRC has proven a consistently accurate and efficient method for correcting sequencing errors in raw long reads (Fu et al. 2019; Zhang et al. 2020). FMLRC2 produces largely identical results to FMLRC albeit with improved speed and stability. In addition to its proven utility for raw error correction, we demonstrate the effectiveness of FMLRC2 as a de novo assembly polisher for diverse prokaryotic and eukaryotic genomes. FMLRC2 consistently outperforms extant genome polishing tools in minimizing residual assembly errors (mismatches and indels) and computational requirements.

## Methods

### FMLRC2

FMLRC2 represents a reimplementation of the original FMLRC from C++ to Rust, without major changes to the underlying algorithm. In benchmark tests, it is about 50% faster than FMLRC (supplementary table S1, Supplementary Material online). FMLRC2 is open source and publicly available at https://github.com/jwanglab/fmlrc2.

### Datasets

We evaluated FMLRC2 against de novo assemblies from 24 bacterial and 6 eukaryotic datasets. Bacterial datasets include four independent datasets from each of six bacterial isolates (*A. baumannii* J9, *C. koseri* MINF_9D, *E. kobei* MSB1_1B, Haemophilus M1C132_1, *K. oxytoca* MSB1_2C, and *K. variicola* INF345) (Wick et al. 2021b). Long-read-only assemblies for each were performed using Trycycler v0.5.0 (Wick et al. 2021a) and Medaka v1.4.3 (https://github.com/nanoporetech/medaka). These data are publicly available at https://doi.org/10.26180/16727680. We additionally used two publicly available sets of ONT sequence data from each of three well-established model eukaryotic organisms: *Saccharomyces cerevisiae* (S288C), *Arabidopsis thaliana* (Columbia; TAIR10.1), and *Drosophila melanogaster* (ISO-1) [table 1]. Experimental sequencing datasets (ONT and Illumina/BGI) were obtained from NCBI (table 2). We simulated ONT data and the corresponding paired-end Illumina dataset using Badread v0.2.0 (Wick 2019) and ART v2016-06-05 (Huang et al. 2011) as previously described (Wick and Holt 2022). Briefly, short reads were simulated using ART with HiSeqX TruSeq preset, to 100X effective sequencing depth, 150 bp read length, 400 ± 50(sd) bp mean fragment. Long reads were simulated using Badread with parameters “–length 20000,12000–identity 90,98,4”. These eukaryotic simulated data are available from Dryad: https://doi.org/10.5061/dryad.gtht76hr3. Basic statistics and coverage of simulated data are described in table 3. FastQC v0.11.9 (Andrews 2010) was used to check for quality issues among experimental short-read datasets. Where necessary, fastp v0.23.2 (Chen et al. 2018) was used to clean the short-read datasets and remove Ns prior to downstream polishing. Long-read-only assemblies were generated for each of the nine experimental and simulated eukaryotic ONT datasets using Flye v2.8.1 (Kolmogorov et al. 2019) followed by Medaka v1.4.3 with default parameters.

**Table 1.** Eukaryotic Model Organisms Used for Assembly Polishing Evaluation.

Species	Strain/Genome	Refseq Accession	Genome Size (Mbp)
*Saccharomyces cerevisiae*	S288C	GCF_000146045.2	12.16
*Arabidopsis thaliana*	Columbia (TAIR10.1)	GCF_000001735.4	119.67
*Drosophila melanogaster*	ISO-1	GCF_000001215.4	143.73

**Table 2.** Experimental Datasets Used for Performance Evaluation.

Species	Sequence Type	Accession Number	Number of Reads	Average Read Length (bp)	Mean Coverage
*Saccharomyces cerevisiae*	ONT	SRR17374240*	105,371	13,726	106×
	ONT	ERR1883398	49,617	8,322	17×
	BGI	SRR17374239	48.6m	150	600×
*Arabidopsis thaliana*	ONT	SRR12136402	2,551,376	4,473	73×
	ONT	SRR16832054**	512,896	27,757	104×
	Illumina	SRR12136403	141m	150	177×
*Drosophila melanogaster*	ONT	SRR13070614	372,834	13,741	34×
	ONT	SRR13070625	640,215	11,142	47×
	Illumina	SRR6702604	41.2m	151	43×

Note.— Seqtk was used to subsample. *15% or **30% of the ONT reads for initial assembly.

**Table 3.** Simulated Datasets Used for Performance Evaluation.

Species	Simulator	Number of Reads	Average Read Length (bp)	Mean Coverage
*Saccharomyces cerevisiae*	Badread (long reads)	19,185	24,841	102×
	ART (short reads)	8.1m	150	100×
*Arabidopsis thaliana*	Badread (long reads)	19,540	24,306	100×
	ART (short reads)	79.6m	150	100×
*Drosophila melanogaster*	Badread (long reads)	16,268	24,162	98×
	ART (short reads)	94.8m	150	100×

### Polishing and Performance Assessment

FMLRC2 v0.1.6 using RopeBWT2 (r187; Li 2014), HyPo v1.0.3 (Kundu et al. 2019), NextPolish v1.4.0 (Hu et al. 2020), ntEdit v1.3.5 (Warren et al. 2019), Pilon v.1.24 (Walker et al. 2014), POLCA v4.0.8 (Zimin and Salzberg 2020), Polypolish v0.5.0 (Wick and Holt 2022), Racon v1.5.0 (Vaser et al. 2017), and wtpoa (Ruan and Li 2019) were used to polish nanopore-only bacterial and eukaryotic assemblies. Each polisher was run once on each assembly using the default parameters, unless otherwise specified. The resulting polished eukaryotic assemblies were then compared against their respective reference genomes using QUAST v5.0.2 (Gurevich et al. 2013). Bacterial assemblies were compared against the respective reference for simulated data or in a pairwise fashion for experimental data as described in Wick and Holt (2022). Briefly, global alignments were computed between polished assemblies and the reference or species-matches assemblies for the simulated and experimental datasets, respectively. Total residual errors—or total pairwise distance—are equivalent to the edit distance, including mismatches and indels. Computational performance (CPU time and memory usage) was determined using “/usr/bin/time -v.” For eukaryote assemblies, Pilon was run four times in succession, showing iterative improvements. As previously shown by Wick and Holt (2022), Pilon rarely produced significant improvement after the first iteration for bacterial assemblies.

## Results

**Fig. 1.**
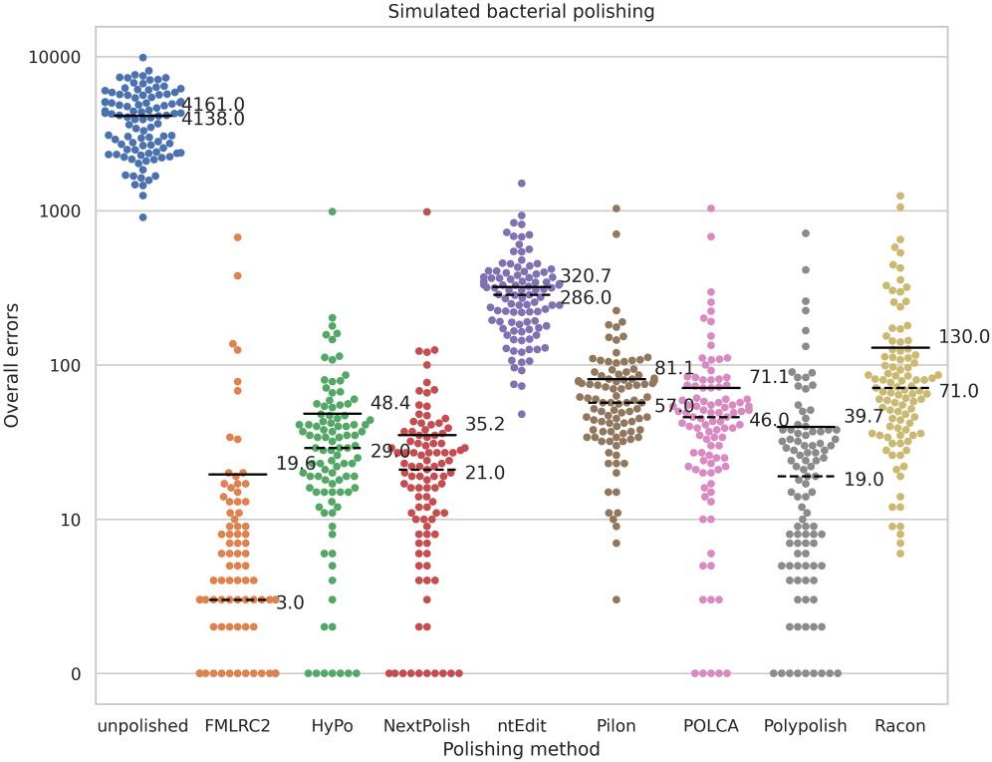
Overall residual errors for polished simulated bacterial genomes. The solid line indicates the mean; dashed indicates the median.

**Fig. 2.**
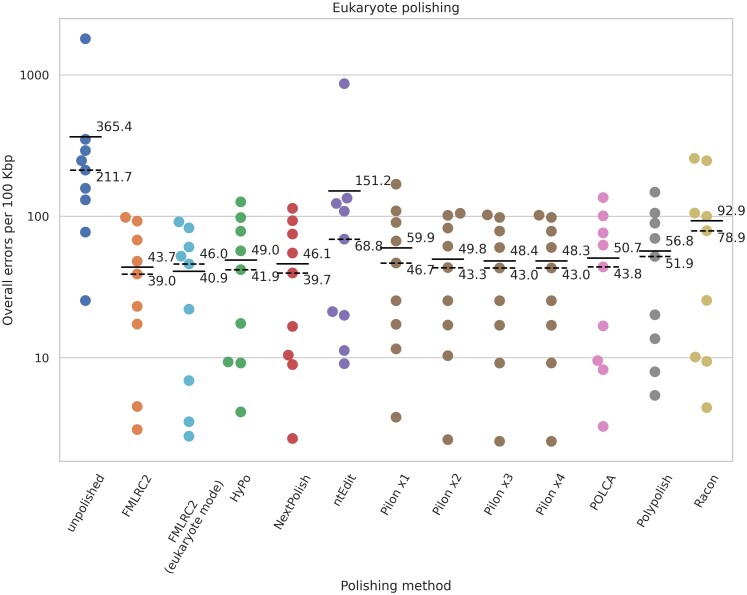
Residual errors per 100 kbp after polishing for experimental and simulated eukaryotic datasets. The solid line indicates the mean; dashed indicates the median.

**Fig. 3.**
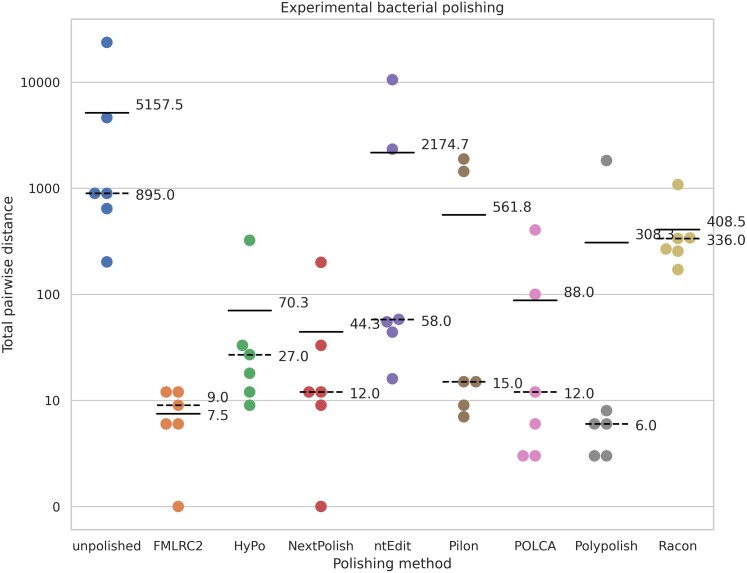
Sum of pairwise differences among replicates from experimental bacterial datasets. The solid line indicates the mean; dashed indicates the median.

We evaluated FMLRC2 against seven other state-of-the-art assembly polishing methods using a combination of simulated and experimental long- and short-read datasets spanning a wide variety of bacterial species and three eukaryotes. Since the ground truth is known for the simulated datasets and reference lines of eukaryotes, we evaluated based on total residual errors among polishing results for simulated bacteria and all eukaryotic datasets (fig. 1 and fig. 2, respectively). Table 4 presents the average residual errors per 100 kbp among simulated and experimental datasets from eukaryotes and the average CPU time and memory usage per polishing run. For experimental bacterial data, we use the total pairwise distance among technical replicates as an indicator of polishing accuracy (fig. 3). Polishing with FMLRC2 results in a dramatically lower residual error and pairwise distance among bacterial datasets. Likewise, it produces the fewest residual errors among eukaryotic datasets, albeit not dramatically lower than the other best-performing methods. However, FMLRC2 requires far less CPU time than the other methods, and 15× faster than the next best-performing method, NextPolish. The memory (RAM) usage is comparable to the other high-performing methods (except ntEdit and wtpoa, which have noticeably poor polishing accuracy).

**Table 4.** Performance of Assembly Polishers Averaged Over Two Experimental and One Simulated Dataset From Each of the Three Species.

Method	Mismatches	Indels	Combined	CPU Time (s)	RAM (GB)
unpolished	65.9	299.5	365.4	−	−
HyPo	29.7	19.4	49.0	32924	29.6
NextPolish	31.4	14.7	46.1	59209	10.5
ntEdit	52.2	99.1	151.2	7185	**1**.**7**
Pilon (x1)	34.5	24.3	58.8	33268	26.2
Pilon (x2)	28.7	21.1	49.8	−	−
Pilon (x3)	28.5	19.9	48.4	−	−
Pilon (x4)	28.5	19.8	48.3	141615	65.6
POLCA	30.5	20.2	50.7	27166	15.7
Polypolish	29.3	27.5	56.8	107703	165.3
Racon	30.1	62.9	92.9	84497	69.3
wtpoa	56.9	127.2	184.1	32061	2.7
FMLRC2 (default)	31.0	**12**.**7**	43.7	**3955**	14.2
FMLRC2 (eukaryote)	**27**.**8**	13.1	**40**.**9**	4387	14.3

Bold values indicate the best performing method for each metric.

Note.—Mismatches and indels represent the average residual errors per 100 kbp. Resource usage was not recorded for intermediate iterations of Pilon after the first, but run time is expected to scale linearly with the number of iterations.

Results of polishing the *Drosophila melanogaster* datasets with FMLRC2 using its default parameters (−branch_factor 4, –cache_size 8, –k 21 59, –min_count 5, –min_frac 0.1) showed relatively poor performance correcting mismatch errors, especially among simulated datasets. We hypothesized that these errors occur in highly repetitive sequences (the likes of which do not exist in most bacterial genomes) when the representation of the true “version” of the repeat falls below the absolute or relative minimum count (min_count and min_frac, respectively). To address this, we evaluated all eukaryotic datasets using alternate parameter settings optimized for resolving these repetitive element problems (−k 21 59 80, –min_frac 0), dubbed “eukaryote mode.” With these settings, the average residual error across eukaryotic assemblies polished with FMLRC2 is further reduced at the cost of a ∼10% increase in CPU time (fig. 2, table 4). Of note, however, FMLRC2 using the *default* settings still outperforms all other evaluated methods.

## Discussion and Conclusion

While the cost of third-generation long-read sequencing, including ONT, continues to decrease, and accuracy increases, hybrid multi-technology methods remain an efficient and effective approach for de novo genome assembly. Following FMLRC's proven performance as an error correction tool for raw reads, we demonstrate FMLRC2's exceptional performance as a polishing tool for de novo nanopore-based assemblies in both bacteria and simple eukaryotes. FMLRC2 outperforms the other tested polishing tools in reducing the residual assembly error, as illustrated using simulated and real ONT sequencing datasets. Assemblies polished with FMLRC2 have the fewest mean residual errors, while FMLRC2 is also the fastest method—over an order of magnitude faster than the next most accurate tool.

## Supplementary Material

msad048_Supplementary_Data

## Data Availability

FMLRC2 is open source and publicly available at https://github.com/jwanglab/fmlrc2. Bacterial data are publicly available at https://doi.org/10.26180/16727680 and simulated eukaryotic data at https://doi.org/10.5061/dryad.gtht76hr3.

## References

[msad048-B1] Amarasinghe S , Su S, Dong X, Zappia L, Ritchie M, Gouil Q. 2020. Opportunities and challenges in long-read sequencing data analysis. Genome Biol. 21:1. 10.1186/s13059-020-1935-5 PMC7006217 32033565

[msad048-B2] Andrews S . 2010. FastQC: A Quality Control Tool for High Throughput Sequence Data [Online]. Available from: http://www.bioinformatics.babraham.ac.uk/projects/fastqc/

[msad048-B3] Chen S , Zhou Y, Chen Y, Gu J. 2018. fastp: an ultra-fast all-in-one FASTQ preprocessor. Bioinformatics 34(17):i884–i890. 30423086 10.1093/bioinformatics/bty560 PMC6129281

[msad048-B4] Fu S , Wang A, Au K. 2019. A comparative evaluation of hybrid error correction methods for error-prone long reads. Genome Biol. 20:1. 30717772 10.1186/s13059-018-1605-z PMC6362602

[msad048-B5] Gurevich A , Saveliev V, Vyahhi N, Tesler G. 2013. QUAST: quality assessment tool for genome assemblies. Bioinformatics 29(8):1072–1075. 23422339 10.1093/bioinformatics/btt086 PMC3624806

[msad048-B6] Hu J , Fan J, Sun Z, Liu S. 2020. NextPolish: a fast and efficient genome polishing tool for long-read assembly. Bioinformatics 36(7):2253–2255. 31778144 10.1093/bioinformatics/btz891

[msad048-B7] Huang W , Li L, Myers J, Marth G. 2011. ART: a next-generation sequencing read simulator. Bioinformatics 28(4):593–594. 22199392 10.1093/bioinformatics/btr708 PMC3278762

[msad048-B8] Jain M , Koren S, Miga K, Quick J, Rand A, Sasani T, Tyson J, Beggs A, Dilthey A, Fiddes I, et al 2018. Nanopore sequencing and assembly of a human genome with ultra-long reads. Nat Biotechnol. 36(4):338–345. 29431738 10.1038/nbt.4060 PMC5889714

[msad048-B9] Kim B , Wang J, Miller D, Barmina O, Delaney E, Thompson A, Comeault A, Peede D, D'Agostino E, Pelaez J, et al 2021. Highly contiguous assemblies of 101 drosophilid genomes. Elife. 10:e66405. 34279216 10.7554/eLife.66405 PMC8337076

[msad048-B10] Kolmogorov M , Yuan J, Lin Y, Pevzner P. 2019. Assembly of long, error-prone reads using repeat graphs. Nat Biotechnol. 37(5):540–546. 30936562 10.1038/s41587-019-0072-8

[msad048-B11] Kundu R , Casey J, Sung W. 2019. HyPo: super fast & accurate polisher for long read genome assemblies. Biorxiv. 10.1101/2019.12.19.882506

[msad048-B12] Li H . 2014. Fast construction of FM-index for long sequence reads. Bioinformatics 30:3274–3275. 25107872 10.1093/bioinformatics/btu541 PMC4221129

[msad048-B13] Michael T , Jupe F, Bemm F, Motley S, Sandoval J, Lanz C, Loudet O, Weigel D, Ecker J. 2018. High contiguity *Arabidopsis thaliana* genome assembly with a single nanopore flow cell. Nat Commun. 9:1. 29416032 10.1038/s41467-018-03016-2 PMC5803254

[msad048-B14] Pollard M , Gurdasani D, Mentzer A, Porter T, Sandhu M. 2018. Long reads: their purpose and place. Hum Mol Genet. 27(R2):R234–R241. 29767702 10.1093/hmg/ddy177 PMC6061690

[msad048-B15] Ruan J , Li H. 2019. Fast and accurate long-read assembly with wtdbg2. Nat Methods. 17(2):155–158. 31819265 10.1038/s41592-019-0669-3 PMC7004874

[msad048-B16] Shi L , Guo Y, Dong C, Huddleston J, Yang H, Han X, Fu A, Li Q, Li N, Gong S, et al 2016. Long-read sequencing and de novo assembly of a Chinese genome. Nat Commun. 7:1. 10.1038/ncomms12065 PMC4931320 27356984

[msad048-B17] Vaser R , Sović I, Nagarajan N, Šikić M. 2017. Fast and accurate de novo genome assembly from long uncorrected reads. Genome Res. 27(5):737–746. 28100585 10.1101/gr.214270.116 PMC5411768

[msad048-B18] Walker B , Abeel T, Shea T, Priest M, Abouelliel A, Sakthikumar S, Cuomo C, Zeng Q, Wortman J, Young S, et al 2014. Pilon: an integrated tool for comprehensive microbial variant detection and genome assembly improvement. PLoS One. 9(11):e112963. 25409509 10.1371/journal.pone.0112963 PMC4237348

[msad048-B19] Wang J , Holt J, McMillan L, Jones C. 2018. FMLRC: hybrid long read error correction using an FM-index. BMC Bioinformatics 19:1. 29426289 10.1186/s12859-018-2051-3 PMC5807796

[msad048-B20] Warren R , Coombe L, Mohamadi H, Zhang J, Jaquish B, Isabel N, Jones S, Bousquet J, Bohlmann J, Birol I . 2019. ntEdit: scalable genome sequence polishing. Bioinformatics 35(21):4430–4432. 31095290 10.1093/bioinformatics/btz400 PMC6821332

[msad048-B21] Watson M , Warr A. 2019. Errors in long-read assemblies can critically affect protein prediction. Nat Biotechnol. 37(2):124–126. 30670796 10.1038/s41587-018-0004-z

[msad048-B22] Wick R . 2019. Badread: simulation of error-prone long reads. J Open Source Softw. 4(36):1316.

[msad048-B23] Wick R , Holt K. 2022. Polypolish: short-read polishing of long-read bacterial genome assemblies. PLoS Comput Biol. 18(1):e1009802. 35073327 10.1371/journal.pcbi.1009802 PMC8812927

[msad048-B24] Wick R , Judd L, Cerdeira L, Hawkey J, Méric G, Vezina B, Wyres K, Holt K. 2021a. Trycycler: consensus long-read assemblies for bacterial genomes. Genome Biol. 22(1):266. 34521459 10.1186/s13059-021-02483-z PMC8442456

[msad048-B25] Wick R , Judd L, Wyres K, Holt K. 2021b. Recovery of small plasmid sequences via Oxford Nanopore sequencing. Microb Genom. 7(8):000631. 34431763 10.1099/mgen.0.000631 PMC8549360

[msad048-B26] Zhang H , Jain C, Aluru S. 2020. A comprehensive evaluation of long read error correction methods. BMC Genomics 21:S6. 10.1186/s12864-020-07227-0 PMC7751105 33349243

[msad048-B27] Zimin A , Salzberg S. 2020. The genome polishing tool POLCA makes fast and accurate corrections in genome assemblies. PLoS Comput Biol. 16(6):e1007981. 32589667 10.1371/journal.pcbi.1007981 PMC7347232

