# Supplementary material for: Polishing De Novo Nanopore Assemblies of Bacteria and Eukaryotes With FMLRC2
Source: Mol Biol Evol. 2023 Mar 3;40(3):msad048. doi: 10.1093/molbev/msad048 (PMC10015616; doi:10.1093/molbev/msad048)
Supplement: msad048_Supplementary_Data [file msad048_supplementary_data.pdf]

SUPPLEMENTARY MATERIAL

Polishing de novo *nanopore assemblies of bacteria and eukaryotes with FMLRC2*

Q. X. Charles Mak, Ryan R. Wick, James Matthew Holt, Jeremy R. Wang.

Taxa	Accession number	FMLRC CPU (s)	FMLRC RAM (GB)	FMLRC2 CPU (s)	FMLRC2 RAM (GB)
S. cerevisiae	SRR17374240	90	4.9	64	6.0
	ERR1883398	199	4.9	96	6.0
	Simulated	75	1.7	64	2.7
A. thaliana	SRR12136402	2,790	27.1	701	28.9
	SRR16832054	3,535	27.2	736	29.0
	Simulated	1,461	16.7	602	18.6
D. melanogaster	SRR13070614	2,051	10.0	943	10.9
	SRR13070625	2,213	10.1	778	11.0
	Simulated	1,219	19.9	796	20.8
Bacteria	Average of 100 samples	18.7	0.58	13.3	1.52
Total		15,500		**6,112**	

Table S1. CPU and memory usage for bacterial and eukaryotic polishing FMLRC and FMLRC2. FMLRC2 takes less than 50% of the total time with a modest increase in memory usage.
